# Magnetic resonance imaging safety in Asia-Oceania: call for action

**DOI:** 10.1007/s11604-024-01729-7

**Published:** 2025-01-18

**Authors:** Rijo Mathew Choorakuttil, Elaine Yee Ling Kan, James Thomas Patrick Decourcy Hallinan, Tamara Razon Cuenza, Evelyn Lai Ming Ho, Rawnak Afrin, Nucharin Supakul, Noriyuki Tomiyama, Chamaree Chupetcharasopon, Danny Hing Yan Cho, Kwan Hoong Ng

**Affiliations:** 1 Department of Preventive Radiology and Integrated Diagnostics, AMMA Scans-AMMA Center for Diagnosis and Preventive Medicine Pvt Ltd, Kochi, Kerala India; 2 Department of Radiology, Hong Kong Children’s Hospital, Kowloon, Hong Kong; 3 Department of Diagnostic Imaging, National University Hospital, Singapore, Singapore; 4 Department of Radiology, Medical Centre Manila, Manila, Philippines; 5 Imaging Department. ParkCity Medical Centre, Kuala Lumpur, Malaysia; 6 Institute of Nuclear Medicine and Allied Sciences, Dhaka Medical College, Dhaka, Bangladesh; 7 Department of Radiology & Imaging Sciences, Indiana University School of Medicine, Indianapolis, USA; 8 Department of Diagnostic and Interventional Radiology, Graduate School of Medicine, Osaka University, Osaka, Japan; 9 MedPark Hospital, Bangkok, Thailand; 10 Department of Diagnostic & Interventional Radiology, Kwong Wah Hospital, Kowloon, Hong Kong; 11 Department of Biomedical Imaging, Faculty of Medicine, Universiti Malaya, Kuala Lumpur, Malaysia

**Keywords:** Awareness, Collaboration, Education, MRI, Safety, Quality assurance

## Abstract

Magnetic Resonance Imaging (MRI) safety is a critical concern in the Asia-Oceania region, as it is elsewhere in the world, due to the unique and complex MRI environment that demands attention. This call-for-action outlines ten critical steps to enhance MRI safety and promote a culture of responsibility and accountability in the Asia-Oceania region. Key focus areas include strengthening education and expertise, improving quality assurance, fostering collaboration, increasing public awareness, and establishing national safety boards. By implementing these actions, we aim to significantly reduce MRI-related incidents and create a culture of safety across diverse healthcare settings in the Asia-Oceania Region.

## Preamble

Magnetic Resonance Imaging (MRI) safety is a critical concern in the Asia-Oceania region, as it is elsewhere in the world, due to the unique and complex MRI environment that demands attention. The potential for safety-related incidents may increase as MRI-based diagnostics and interventions become more widely used and scanners with higher field strengths and capabilities are deployed. However, MRI safety incidents [1, 2] are grossly under-reported, and the system must become robust to recognise and address MRI safety issues. It is essential to report and learn from incidents, understand their causes, and take prompt action to prevent similar incidents in the future that can minimise avoidable human suffering and save hospital and litigation costs. MRI technologists, radiographers and radiologists must know the rapidly evolving changes in MRI safety practices to optimise patient safety [3]. The negative environmental impact of MRI which has relatively high greenhouse gas (GHG) emissions has now been recognised [4] and steps taken to incorporate sustainable practices will also benefit personnel and patient safety. All these factors highlight the need for collective action from all the stakeholders mentioned earlier, plus medical physicists, biomedical engineers, and the industry at large.

This call-for-action outlines ten critical steps to enhance MRI safety and promote a culture of responsibility and accountability in the Asia-Oceania region. Key focus areas include strengthening education and expertise, improving quality assurance, fostering collaboration, increasing public awareness, and establishing national safety boards. By implementing these actions, we aim to significantly reduce MRI-related incidents and create a culture of safety across diverse healthcare settings in the Asia-Oceania Region.

## MRI safety: call for action

To address the growing concern of MRI safety, AsiaSafe developed this MRI safety ten-point call for action

### Increase educational initiatives

- Enhance education programs for healthcare professionals, focusing on MRI safety principles, protocols, and best practices. Develop modular educational programs that can be learnt in phases leading to a more comprehensive education. The modular education approach will allow personnel to pick and choose modules that are relevant to their practice based on their current educational and clinical experience. Modular education will also integrate hands-on applications in real life for an immersive experience.
- Establish a strong knowledge base through education programs, focusing on MRI safety principles, protocols, and best practices. The focus will be on establishing a database of trainers and trainees and a system for regular mentoring and feedback to enhance the knowledge base and pool of knowledgeable personnel.
- Develop structured educational pathways for new learning, refresher learning and upskilling. These pathways will include education for new entrants into the MRI domain (medical and non-medical) and cover clinical and administrative aspects relevant to MRI safety and environment. These pathways will also be aimed at allowing MRI personnel to step up into roles of greater responsibility with clear career progression.
- Design, develop and promote multimedia resources for MRI safety education. These will include conventional narrative or textual materials such as articles, lecture handouts, books, audio, video, animation, art or sketch resources and interactive models on MRI settings. These resources can serve as a digital library for on-demand use by MRI-related personnel.
- Develop clinical and administrative protocols to update educational resources with up-to-date scientific evidence. These will include framing the policies and regulations that guide the review and updating of existing clinical and administrative protocols and lead from evidence and performance and critical incident audits.
- Develop a dedicated online portal and repository for MRI safety educational resources. The online portal can serve as an on-demand digital library accessible by all MRI personnel. This will prove to be especially valuable for more remote areas and regions within Asia-Oceania which have relatively limited access to MRI safety training and resources.

### Elevate expertise: organize continuous education, training and certification

- Provide easily accessible informational and educational tools and resources. These materials may consist of free and downloadable references including manuals, policies, guidelines, protocols, algorithms, and updates related to MRI safety for the orientation and guidance of administrators and staff working in MRI environments.
- Provide regular structured training sessions and workshops for healthcare professionals with an emphasis on MRI safety issues including advanced education for MRI personnel and job specific training for non-MRI personnel ensuring competence in safety within the MRI environment. Apart from initial training, annual or semi-annual retraining should be required to reinforce MRI safety proficiency..
- Develop online training modules for healthcare professionals that can be accessed sequentially or in full, targeting both primary and continuing MRI safety education. The dedicated online portal and repository mentioned in Call for Action No. 1 should ensure a modular educational approach that includes primary and continuing MRI Safety education for healthcare professionals.
- Develop certification standards for the specific roles and responsibilities of MRI professionals in line with international consensus recommendations. Ideally, every MRI diagnostic or research facility in Asia-Oceania should have specially assigned personnel who have completed certification training in the roles of MRI Medical or Research Director, MRI Safety Officer, and MRI Safety Expert. This ensures leadership and a high level of expertise in MRI safety within every MRI environment. In addition, protocols should be developed for reassessment and recertification of MRI professionals to maintain competency and qualifications.
- Continuous education and training should include awareness and promotion of environmentally sustainable MRI practices, which directly and indirectly contribute to patient safety and the larger healthcare and climate spheres.
- Disseminate important updates on MRI Safety issues as they evolve.

### Enhance public awareness and education

- Raise awareness among patients, their families, and the public about MRI safety risks and benefits to ensure informed decision-making. This includes determining whether the MRI examination is the most appropriate for them, whether there is an absolute need for contrast administration, and whether the sequences or MRI protocol are optimized and as short as possible to reduce time in the MRI scanner. Emphasize the importance of informing healthcare providers about any implanted devices
- Organise and host regular awareness sessions (face-to-face or online) on MRI safety with different stakeholders including patients, families and the public. These sessions will highlight preventive steps that can be adopted at various levels of the MRI environment. They will also provide checklists that all stakeholders involved in MRI can use to improve safety. A core concept is to integrate patients and families as important stakeholders in MRI safety and to increase awareness so that they are equally involved and responsible for their safety in an MRI environment.
- Regularly organise and host dialogue sessions with all stakeholders focused on interactive feedback and constructive development of safer practices. Involving them in formalized committees whether at the hospital, local or national level will ensure not only buy-in but equal involvement and responsibility for MRI safety.
- Create and maintain dedicated social media handles across different platforms to disseminate relevant information on MRI safety. Establish a social media team responsible for creating, collating, reviewing, and disseminating content based on evidence and best practices. Develop policies for performance reviews, team changes or rotations, and periodic evaluation of social media handles.
- Create a publicly accessible digital archive of MRI safety-related educational and awareness resources for all stakeholders involved with MRI safety and the MRI environment.
- Create multilingual awareness materials to address the linguistic diversity in Asia-Oceania.

### Encourage multidisciplinary collaboration

- Multidisciplinary collaboration in MRI safety is highly effective as it combines the expertise of various professionals to address and mitigate safety threats. Continuous quality improvement projects should be conducted across institutions in the Asia-Oceania to identify and address gaps in MRI safety practice. These projects must involve stakeholders from multiple disciplines, including radiologists, technologists, physicists, engineers, other healthcare professionals and industry professionals, to effectively mitigate safety threats and transform near misses into never events. Such efforts should culminate in publications which can be quoted and shared effectively.
- Establish frameworks for such collaborations in quality improvement and audits and to integrate feedback into existing practice. These frameworks should incorporate feedback from all stakeholders, ensuring that the practices are continuously refined.
- Identify essential and good-to-have stakeholders as part of the multiple disciplines team. A mechanism to collect perspectives from all stakeholders [5], will ensure that all relevant viewpoints are considered, leading to comprehensive safety protocols.
- Implementation of comprehensive MRI safety programs in Asia-Oceania should educate all personnel, including ancillary staff such as housekeeping and anaesthesia technologists. By incorporating these approaches, institutions can ensure that every staff member, regardless of their role, is well-informed and compliant with safety protocols, thereby minimizing the risk of accidents. The education should be conducted periodically – at least twice a year, and every time new staff come on board in the medical facility.
- Create inclusive ‘all-stakeholder’ forums for dedicated discussions, facilitating regular exchanges of ideas, innovative solutions and problems. Such discussion forums or platforms must include the patient voice. MRI safety issues can be discussed regularly (including workshops or special interest group meets) at all conferences/meetings at national, regional and international levels.

### Promote knowledge and experience sharing

- Promote dedicated online portals for technical professionals, clinicians, support staff, and the general public to share MRI safety knowledge.
- Incident reporting is crucial for learning from errors and preventing similar incidents. An anonymised online portal for sharing incidents, the resolution process and how the incident can be prevented in the future should be developed. Locally, it helps improve practices, policies, and procedures, while nationally, it identifies emerging risks. Incidents should be reported through local systems, and the risk management team will handle further reporting to relevant agencies at the national and regional level. All incidents must also be reported to the device manufacturer to determine if there are any equipment faults.
- Organize regular educational webinars and workshops where experts can present their latest research and contribute their experiences and innovations for MRI safety.
- Systematically document and share successful safety practices and lessons learned from past incidents to contribute to the development of local, and eventually regional, safety manuals that address regional contexts and challenges in implementation.
- Provide an online portal for MRI practitioners (technologists/ radiographers, radiologists) to share challenging scenarios, implants encountered or MRI equipment-specific capabilities. This would provide a useful support system in addition to the formal educational, training and certification channels.

### Strengthen quality assurance programs

- Enhance quality assurance programs to ensure compliance with MRI safety standards, guidelines, and regulations. These programs must include identifying relevant stakeholders in the quality assurance pathway and outlining the critical steps involved. They should clearly formulate best practices and define quality standards at each step. Specify who is responsible for each action, establish abort or exit criteria, and create pathways to inform or provide feedback on quality failures. Additionally, determine quality check audit intervals, components, and interpretations of the quality assurance process
- Adopt industry software and hardware enhancements for safer MRI scans with continuous feedback for continued improvement.
- Develop and adapt protocols and guidelines on MRI safety that demarcate roles and responsibilities at each level of the MRI environment.
- Develop an audit system that focuses on identifying limitations, improving systems and integrating this information into educational and training modules.
- Develop incident reporting formats, databases, and pathways to enable regular analysis and interpretation of this information

### Practice effective communication

- Promote clear and effective communication between healthcare professionals, patients, and families regarding procedures, safety, and risks within the MRI environment. Develop standardized systems and methods for the adequate and reliable transmission of MRI safety information and guidelines in simplified, understandable, and concise formats. Information can be shared strategically using various methods, including signs, posters, brochures, and algorithms targeted to different groups to ensure comprehension and minimize risk.
- Involve the ‘patient voice’ through patient and public representatives in the development of such information and guidelines. Designing posters with self-explanatory graphics for patients and their families, placed in strategic locations within healthcare facilities and imaging departments, may be a practical option for Asia-Oceania, where there is a diversity of cultures and languages. Special consideration should be given to the pediatric population undergoing MRIs, as specialized needs arise, including the potential need for sedation with additional attendant risks.
- Advocate for attitudes of openness and cooperation among team members performing various functions within the MRI environment. Recognize that safety gaps at the point of care may result from communication breakdowns, especially during staff shift changes or emergencies. To foster an effective team approach to MRI safety, properly address questions, concerns, near misses, and adverse events through joint discussions and meetings.
- Promote a safe MRI environment for patients and staff by emphasizing a culture of safety nurtured by effective communication and collaborative teamwork.

### Nurture a safety and no-blame culture

- Actively encourage an environment where open communication and transparency are the norms at institutions across the Asia Oceania region. By fostering a culture that supports the reporting and discussion of MRI safety concerns without fear of retribution, institutions can identify and address safety issues more effectively. This approach helps ensure that safety protocols are continuously improved and adapted to new challenges.
- Promote true teamwork where everyone is looking out for each other and the patient, sharing the same goals of safe MRI practice. This should highlight that the team relies on each other and functions as one, where all members are equally responsible when things go right or wrong. Such teams have reduced stress levels and provide higher quality patient care [5, 6].
- Encourage MRI facilities to conduct regular safety drills and simulations tailored to their specific environments. These exercises should be led by designated MRI safety personnel and involve staff from various departments, particularly those responsible for high-risk areas [7]. These drills are crucial for reinforcing safe practices and ensuring that staff are well-prepared for emergency situations.
- Advocate along with other national and regional organisations for the integration of advanced technologies, such as artificial intelligence including natural language processing, into MRI safety protocols. These technologies can significantly enhance safety by identifying patients with contraindicated implants and other high-risk factors before they enter the MRI suite and assist the MRI technologists/radiographers in the adjustments needed before scanning the patient. Implementing AI-driven systems can help institutions manage risks more effectively and ensure that MRI procedures are conducted safely. However, excessive reliance on artificial intelligence must be avoided.
- Incorporate sustainable safety practices that help to reduce greenhouse gas (GHG) emissions. This includes using AI applications and appropriate use indications, including more efficient protocoling to answer a clinical question and even lower strength MRI scanners which can produce high quality diagnostic images and guide intervention. Reducing the need for gadolinium through software enhancements and advanced MRI sequences would decrease gadolinium contamination of waterways [3, 8] and eliminate the risk associated with the unknown long-term impact of gadolinium retention in the body, even though, to date, no adverse effects of retained gadolinium have been reported [9, 10]. Other potential adverse reactions such as nephrogenic systemic fibrosis are also avoided. [11, 12]
- Promote a proactive and collaborative MRI safety culture by encouraging institutions to collaborate and share best practices. Leveraging technology and regularly updating safety protocols based on the latest research and innovations are key components of this approach.

### Monitor advancements in interventional procedures

- Engage stakeholders, including interventional radiologists, healthcare professionals from surgical disciplines, MRI physicists, technologists, anaesthesiologists and industry partners to encourage and promote advances in interventional radiology and surgical procedures that are safe in the MRI environment.
- Identify potential hazards and risks associated with intraoperative MRI and MRI interventions and develop safety guidelines and workflows to address them.
- Develop a database platform that includes intervention incident reporting and improvement measures that are meaningful and accessible to all stakeholders, including industry partners. This aligns with Call-to-Action No. 5.
- By adopting these measures, we can ensure MRI safety in Asia-Oceania and protect patients, healthcare professionals, and the general public from the potential risks associated with MRI procedures.

### Establish national MRI safety boards / committees

- Establish a national MRI safety board to oversee MRI safety initiatives, provide guidance, ensure accountability, and potentially act as an accrediting MRI safety body. The board would be able to advance MRI safety initiatives at health ministries and governmental levels and could be involved in legislative efforts to ensure MRI safety is prioritized.
- The Safety Board will oversee:
  o Development of guidelines and checklists for certification of training, audits, and quality assessment protocols.
  o Development of specific workflows and patient safety protocols for all stages of interaction with the MRI process, including pre-imaging, imaging, and post-imaging stages.
  o Development of protocols for identification of risk factors, screening, precautions for special patient populations (e.g., pediatrics), management of adverse events, emergency response protocols, and critical incident reporting.
  o Development of standard operating protocols to report critical incidents and the database structure to document critical incidents.

## Conclusion

The implementation of these ten action points is crucial for advancing MRI safety in the Asia-Oceania region. By focusing on education, collaboration, and systematic improvements, we can create a safer MRI environment for patients and healthcare professionals alike. The success of this initiative depends on the concerted efforts of all stakeholders in the MRI community across Asia-Oceania.
